# Supplementary figures and images for: Repression of the Hox gene abd-A by ELAV-mediated Transcriptional Interference
Source: PLoS Genet. 2021 Nov 15;17(11):e1009843. doi: 10.1371/journal.pgen.1009843 (PMC8629391; doi:10.1371/journal.pgen.1009843)

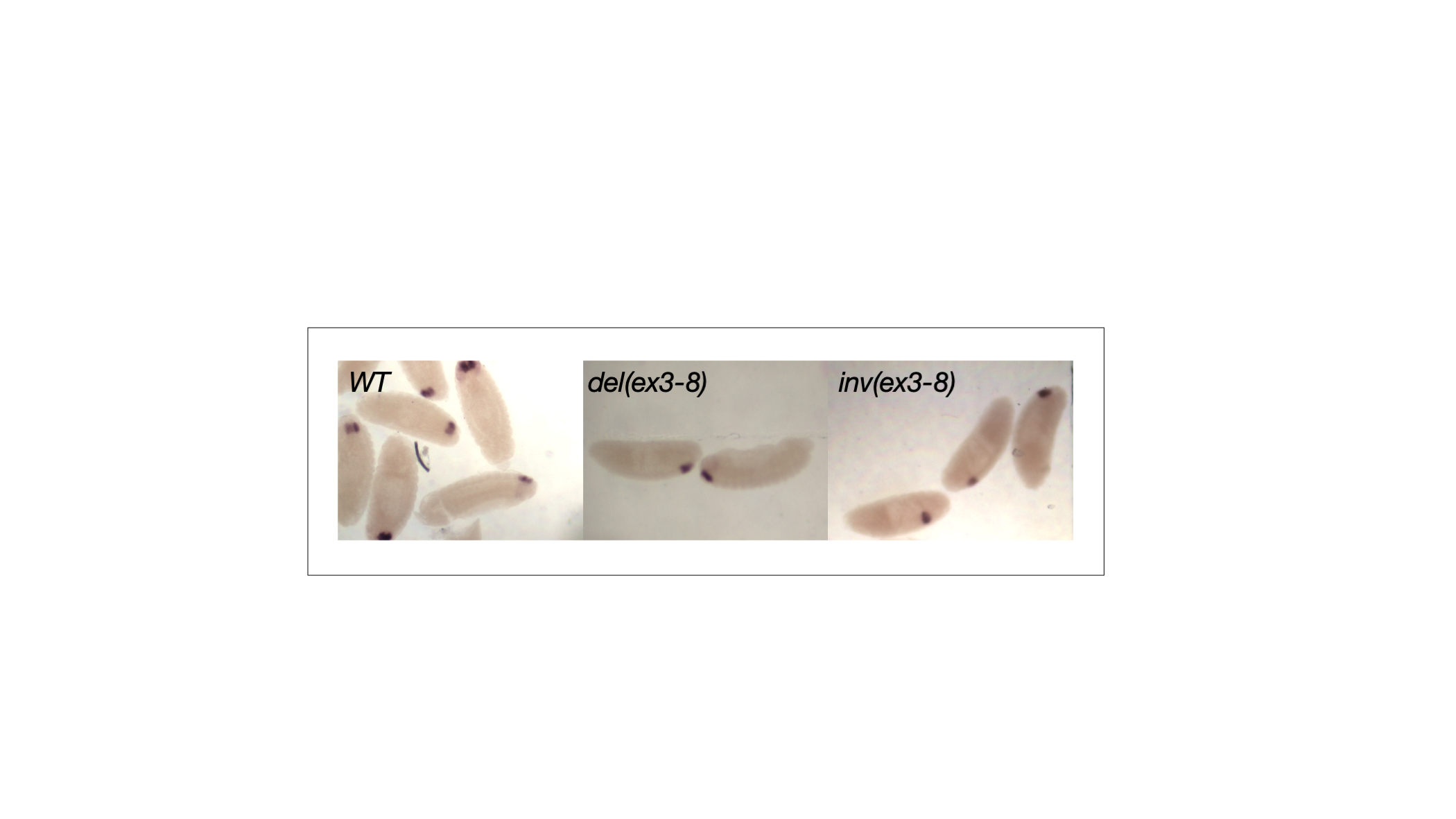

Supplement: S1 Fig — Collections of wild-type (WT) embryos, del(ex3-8) embryos and inv(ex3-8) embryos (genotypes marked on figure) are shown, stained with a probe to exons 1 and 2. (TIFF) [file pgen.1009843.s001.tiff]

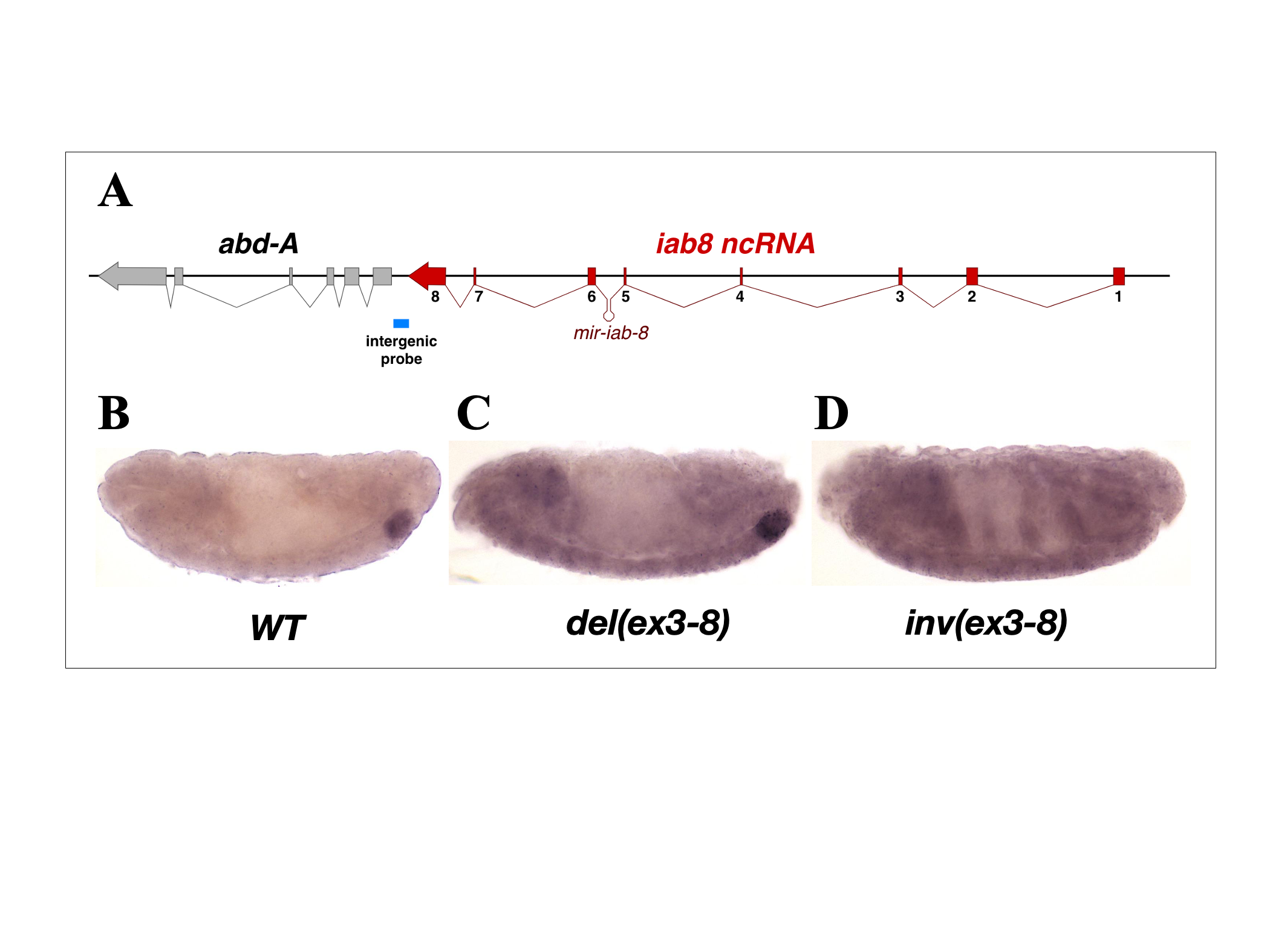

Supplement: S2 Fig — A. Shows the genomic region with the location of the probe marked by a blue bar beneath the map. Stage 13/14 embryo from a wild-type (B.), del(ex3-8) (C.) and inv(ex3-8) (D.) embryos. Expression in the posterior CNS can be seen in wild-type (B.) and del(ex3-8) embryos (C.) but not in inv(ex3-8) (D.) embryos. (TIFF) [file pgen.1009843.s002.tiff]

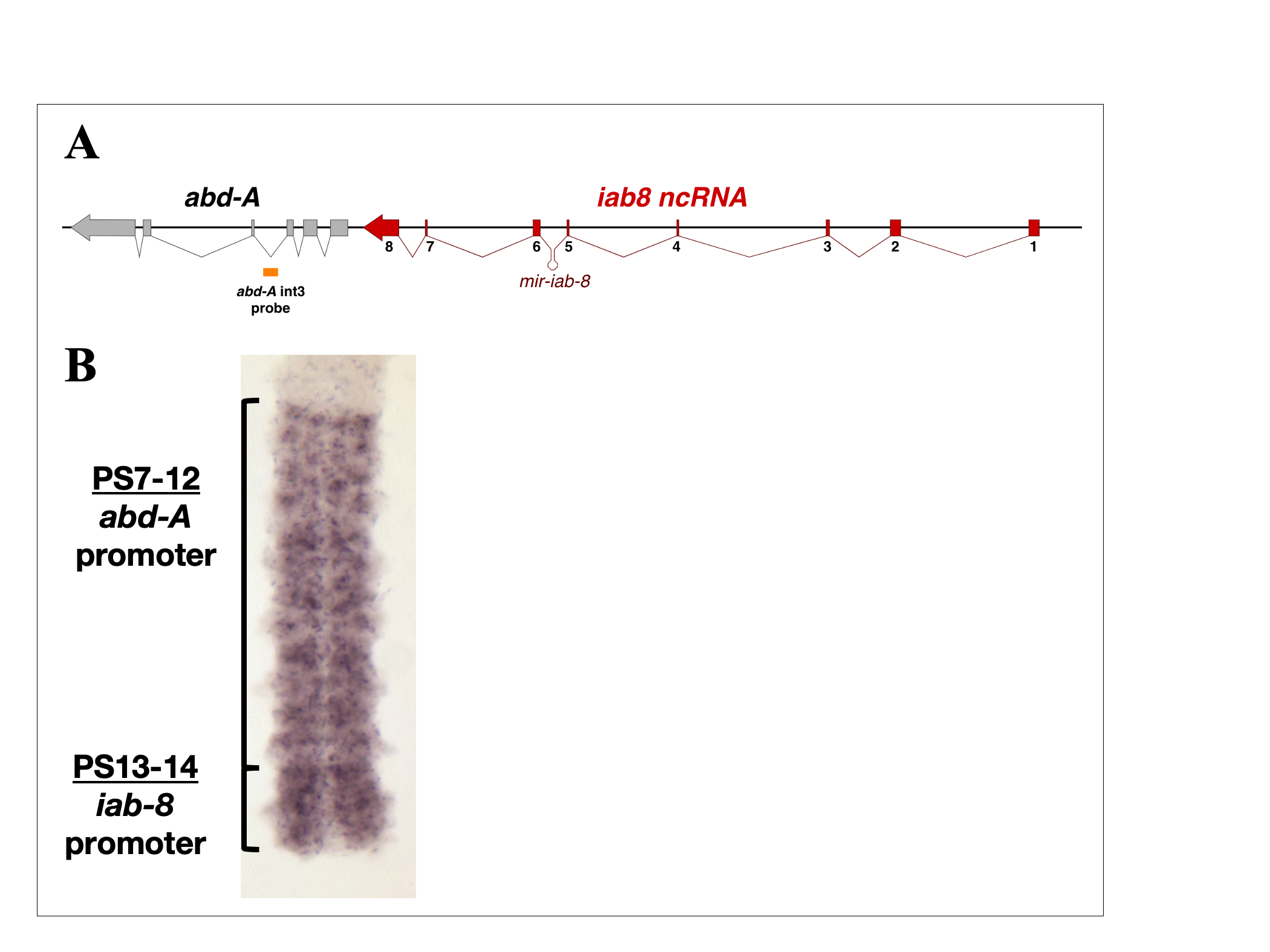

Supplement: S3 Fig — Similar rates of transcription of the abd-A and iab-8 ncRNA transcription units are revealed in the CNS with the help of an intronic probe derived from intron 3 of the abd-A gene (indicated by the orange rectangle below the genomic map in A). B, note that while the patterns of expression detected from P7 to 12 is generated by the abd-A promoter, the pattern detected in PS13 and 14 originates from the iab-8 ncRNA. (TIFF) [file pgen.1009843.s003.tiff]

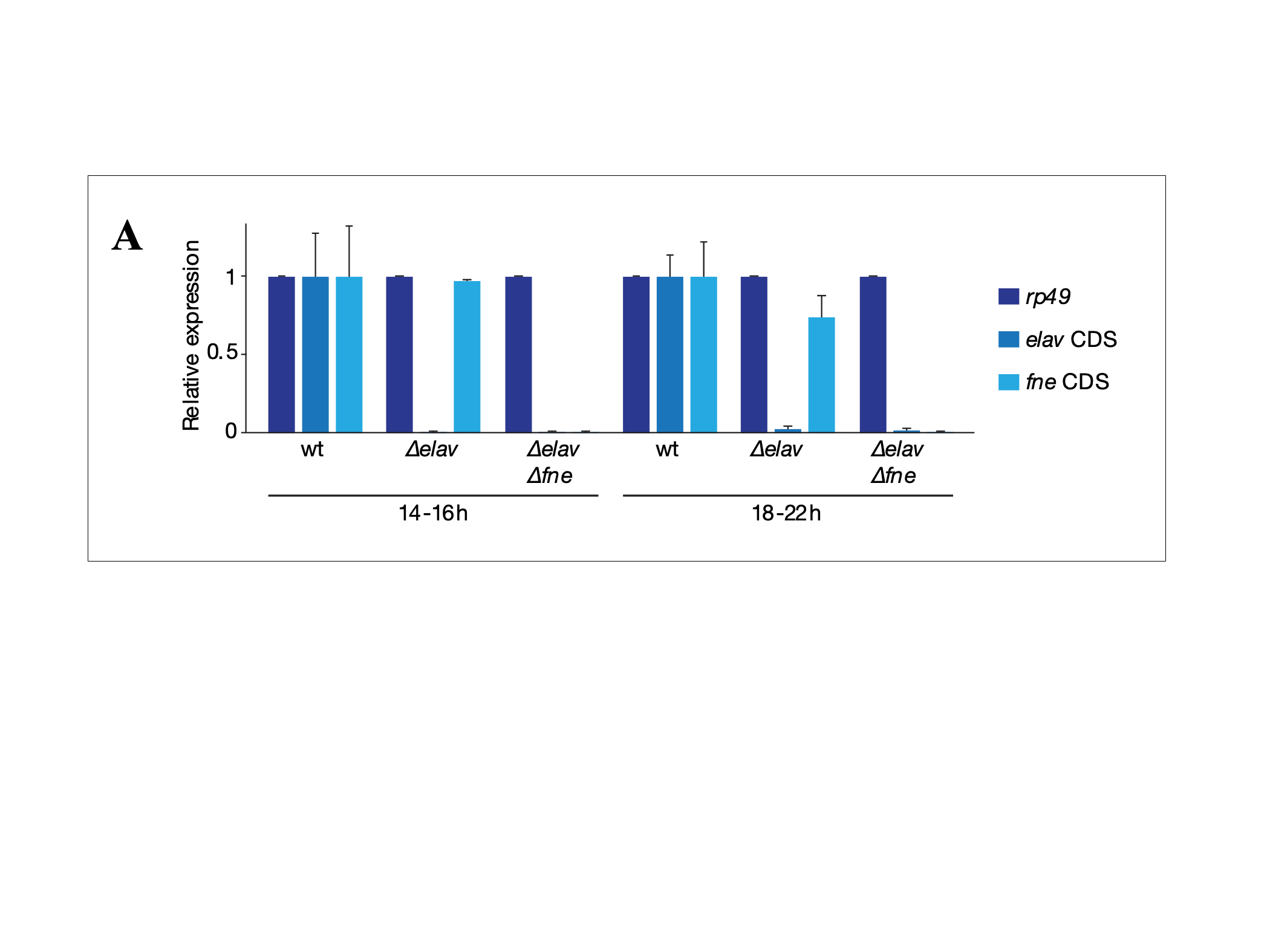

Supplement: S4 Fig — RT-qPCR quantification of elav and fne coding sequence (CDS) RNAs in wild-type, Δelav, and Δelav Δfne embryos. RNA was extracted from whole embryos 14-16h AEL (after egg laying) and 18-22h AEL. RNA levels were normalized to RpL32 (rp49) mRNA levels. Error bars represent mean ± SD of three biological replicates (10–15 embryos per replicate) for each genotype. (TIFF) [file pgen.1009843.s004.tiff]
