# Supplementary material for: Repression of the Hox gene abd-A by ELAV-mediated Transcriptional Interference
Source: PLoS Genet. 2021 Nov 15;17(11):e1009843. doi: 10.1371/journal.pgen.1009843 (PMC8629391; doi:10.1371/journal.pgen.1009843)
Supplement: S1 Table — (DOCX) [file pgen.1009843.s005.docx]

**S1 Table.**

| *pUASt UAS Fw* | GCTAGCGGATCCAAGCTTGC |
| --- | --- |
| *pUASt UAS Rv* | GCGGCCGCAGATCTGTTAAC |
| *PYexC1GFPFw* | AGAGTTAAAAAACGAAAAGAGGCATTTTATTTCGCAGAACAAGC |
| *PYex8C1ex8Fw* | AAGCGGTCTCCCCCAAAATGCCTAAGAAGAAGAG |
| *PYex8C1GFPRv* | CTTAGGCATTTTGGGGGAGACCGCTTTACGC |
| *PYex8C1ex8Rv* | GCTCGAGAGATTACAAACGGAG |
| *PYex8C1ex8Rv* | CAGCTCCGTTTGTAATCTCTCG |
| *PYex8C1intRv* | CCTGCAGGAGTCGACGGT |
| *iab8nc* *Ex6-7F* | CACCAGCTACAACAAAGGAC |
| *iab8* *ex8rev* | CTACCAGTTAATGTGCTTCCTACCTGTC |
| *abdA Ex5R* | GTAACTCCTTCTTCAGCTTCAT |
