# Supplementary material for: Repression of the Hox gene abd-A by ELAV-mediated Transcriptional Interference
Source: PLoS Genet. 2021 Nov 15;17(11):e1009843. doi: 10.1371/journal.pgen.1009843 (PMC8629391; doi:10.1371/journal.pgen.1009843)
Supplement: S2 Table — (DOCX) [file pgen.1009843.s006.docx]

**S2 Table.**

| *attBexon8flk3'* | TAGGGATAACAGGGTAATGTACCGCGTCGACGATGTAGGTCACGGTCTCGAAGCCGCGGTGCGGGTGCCAGGGCGTGCCCTTGGGCTCCCCGGGCGCGTACTCCACCTCACCCATCTGGTCCATCATGATGAACGGGTCGAGGTGGCGGTAGTTGATCCCGGCGAACGCGCGGCGCACCGGGAAGCCCTCGCCCTCGAAACCGCTGGGCGCGGTGGTCACGGTGAGCACGGGACGTGCGACGGCGTCGGCGGGTGCGGATACGCGGGGCAGCGTCAGCGGGTTCTCGACGGTCACGGCGGGCATGTCGACCCTCTTCTACTATTGCTCTCATCCTCCTTCGTAGCGCAAATGTGTGCGGAAATAAAGTGAATCTGCAATTTCTTAACTTTTCTACTTAGAGAACTTTGCTATCTGACATTTCGAAGGATTTTACACAGAGAGTTAAAAAACGAAAAGAGGCAT |
| --- | --- |
