# Supplementary material for: Repression of the Hox gene abd-A by ELAV-mediated Transcriptional Interference
Source: PLoS Genet. 2021 Nov 15;17(11):e1009843. doi: 10.1371/journal.pgen.1009843 (PMC8629391; doi:10.1371/journal.pgen.1009843)
Supplement: S3 Table — (DOCX) [file pgen.1009843.s007.docx]

**S3 Table.**

| **Name** | **F primer** | **R primer** | **Hybridization temperature** |
| --- | --- | --- | --- |
| *abdA:GFP* | CAGCCGTCGAAGTCGGGAGGC | CCATGCCATGTGTAATCCCAGCAGCC | 65°C |
| *iab8 ex1-2* | GGAGTCGCATTCGAAGTCTG | CCGTCGGCCTTTGTTTCTGC |  |
| *iab8 ex8* | CGCTCGAGAGATTACAAACG | GGTGTATTACGGTCAAGGGGG |  |
| *intergenic* | GTGCTGTCTTTTTGCCGAGTCGGC | GGCTCACTCCCTGACTCAACGTCG |  |
| *abdA 3'UTR* | GCTGACAACCACCCATCGCCC | CCCCTTGGCTGAAATCTGTTTGCATG |  |
| *GFP (FM7,act:GFP)* | CACTGGAGTTGTCCCAATTC | CATGCCATGTGTAATCCCAG |  |
| *vir iab8ex8* | CTTTCGGTCCTATTCAACGG | CCGATCCTGCTGGTGTC |  |
| *vir abdA 3'UTR* | GCGACCGAGAGGAACAAGAG | CCAATCATTGTTGCCAGTCTGG |  |
