# Supplementary material for: Repression of the Hox gene abd-A by ELAV-mediated Transcriptional Interference
Source: PLoS Genet. 2021 Nov 15;17(11):e1009843. doi: 10.1371/journal.pgen.1009843 (PMC8629391; doi:10.1371/journal.pgen.1009843)
Supplement: S4 Table — (DOCX) [file pgen.1009843.s008.docx]

**S4 Table.**

| **Name/Target** | **Sequence** |
| --- | --- |
| *intergenic region fwd* | gacagcactgcctcttctttt |
| *intergenic region rev* | aaataaaggcgcacaaaaggc |
| *iab8 ncRNA F* | aggtactcagccactcacag |
| *iab8 ncRNA R* | cccaccgcttatagtccaca |
| *rp49 fwd* | gcaccaagcacttcatcc |
| *rp49 rev* | agcggcgacgcactctgt |
| *fne CDS fwd* | cgaaccgatcaccgtcaagt |
| *fne CDS rev* | ttgagccttggcgctattg |
| *elav cds fwd* | catgcacacgccagttaacaa |
| *elav cds rev* | gtcgcccgccattggt |
