# Supplementary material for: Repression of the Hox gene abd-A by ELAV-mediated Transcriptional Interference
Source: PLoS Genet. 2021 Nov 15;17(11):e1009843. doi: 10.1371/journal.pgen.1009843 (PMC8629391; doi:10.1371/journal.pgen.1009843)
Supplement: S5 Table — (DOCX) [file pgen.1009843.s009.docx]

**S5 Table.**

| **Antibody** | **Manufacturer** | **Dilution** |
| --- | --- | --- |
| Mouse anti-ELAV | DSHB | 1:1000 |
| Goat anti-ABD-A | Santa Cruz Biotechnology | 1:50 |
| Mouse anti-ABD-A | Santa Cruz Biotechnology | 1:200 |
| Rabbit anti-GFP | Torrey Pines Biolabs | 1:1000 |
| Mouse anti-EN | DSHB | 1:100 |
| Guinea pig anti-DPN | kindly provided by G.Technau | 1:1000 |
