# Supplementary material for: Repression of the Hox gene abd-A by ELAV-mediated Transcriptional Interference
Source: PLoS Genet. 2021 Nov 15;17(11):e1009843. doi: 10.1371/journal.pgen.1009843 (PMC8629391; doi:10.1371/journal.pgen.1009843)
Supplement: S6 Table — (DOCX) [file pgen.1009843.s010.docx]

**S6 Table.**

| 12653477 | 12655128 | abdA splice junction intron 1 |
| --- | --- | --- |
| 12662307 | 12663366 | iab8 intron 4 |
| 12665823 | 12667591 | iab8 intron 4 |
| 12669438 | 12671067 | iab8 intron 4 |
| 12693001 | 12694424 | iab8 intron 2 |
| 12699642 | 12700909 | iab8 intron 2 |
| 12702188 | 12703519 | iab8 intron 2 |
| 12723211 | 12724386 | iab8 intron 1 |
| 12737853 | 12739488 | iab8 intron 1 |
| 12751825 | 12754538 | AbdB 3'UTR |
