# Supplementary material for: Repression of the Hox gene abd-A by ELAV-mediated Transcriptional Interference
Source: PLoS Genet. 2021 Nov 15;17(11):e1009843. doi: 10.1371/journal.pgen.1009843 (PMC8629391; doi:10.1371/journal.pgen.1009843)
Supplement: S7 Table — (DOCX) [file pgen.1009843.s011.docx]

**S7 Table.**

| 12653392 | 12655380 | abdA intron 1 |
| --- | --- | --- |
| 12655872 | 12657586 | iab8 exon8 + intergenic region |
| 12657590 | 12658993 | iab8 exon 8 |
| 12661860 | 12663572 | iab8 intron 6 |
| 12665758 | 12667658 | iab8 intron 6 |
| 12668872 | 12670921 | iab8 intron 6 |
| 12694942 | 12696321 | iab8 intron 4 |
| 12699560 | 12700930 | iab8 intron 3 |
| 12723343 | 12725024 | iab8 intron 2 |
| 12734188 | 12735032 | iab8 intron 1 |
| 12738136 | 12739498 | iab8 intron 1 |
| 12751807 | 12754516 | AbdB 3UTR |
