# Supplementary material for: Repression of the Hox gene abd-A by ELAV-mediated Transcriptional Interference
Source: PLoS Genet. 2021 Nov 15;17(11):e1009843. doi: 10.1371/journal.pgen.1009843 (PMC8629391; doi:10.1371/journal.pgen.1009843)
Supplement: S1 Text — (DOCX) [file pgen.1009843.s012.docx]

**Supplementary Materials and Methods**

### Fixation and fluorescence immunostaining of *Drosophila* embryos

Embryos were collected overnight on agar plates. In the morning after, the embryos were dechorionated by immersion for 2min in commercial bleach, and fixing them for 20min in a glass tube with 1mL of 3.6% formaldehyde in antibody staining fixation buffer and 1mL of heptane. After fixation, the embryos were devitellinized by replacing the fix solution with by 1mL of ice-cold methanol, and vortexing for 1min. The devitellinized embryos, which fall to the bottom of the tube, are washed 3 times with methanol, followed by one wash with PBT.

After the fixation, the embryos are blocked by performing 3 quick washes and 3 washes of 20min in PBTX (PBT with 0.1% BSA). After the blocking, the corresponding primary antibodies diluted in PBTX (see table X) are added and incubated overnight.

The morning after, the embryos are subjected to 3 quick washes and 3 washes of 20min in PBTX, and the secondary antibodies are added and incubated for 1h30min at room temperature in the dark. After the incubation, the embryos are again subjected to 3 quick washes and 3 washes of 30 minutes in PBTX. The embryos are then mounted on a slide using glass coverslips as spacers to prevent flattening, or are dissected with tungsten needles and the central nervous systems are mounted on slides. In both cases the embryos are mounted in Vectashield Antifade Mounting Medium (Vector Labs) with DAPI.

### RNA *in situ* hybridization of *Drosophila* embryos

#### Synthesis of DIG-labelled RNA probes for in situ hybridization

Regions to be probed are amplified by PCR and cloned into the vector pGemT Easy (Promega). 4μg of the vector containing the probe sequence is then linearized by cutting with a restriction enzyme that leaves a 5’overhang or a blunt end. The cut plasmid is then treated with proteinase K (Promega) in a total volume of 100μL following the manufacturer’s instructions, and then subjected to a phenol/chloroform extraction to further eliminate any possible protein contaminant from his DNA template.

1μg of the purified template is then used to to synthesize the RNA probe using the *DIG RNA Labelling Kit (T7/SP6)* (Roche) following the manufacturer’s instructions. In order to verify the synthesized product, 1μL of the freshly produced RNA probe is run on a 1% agarose gel. The remaining probe is then precipitated with LiCl precipitation and resuspended in 100μL of DEPC- treated ddH_2_O.

#### RNA *in situ* hybridization using DIG-labelled probes and AP staining

Embryos are collected and fixed using a similar protocol to that used for immunostaining, with the main modification being that the fixative solution is 4% paraformaldehyde in PBS. The embryos are then stored in methanol at -20°C at least for one week before using them.

Fixed embryos are then washed twice in 100% ethanol, and then rotated for 1h in a 1:1 ethanol:xylene solution at 4°C for permeabilization. After this, the embryos are again washed twice in ethanol, then once in methanol. The embryos are then rehydrated by doing washes of 5min in a series of dilutions of methanol/ddH_2_O (100% - 75% - 50% - 25% - 0%). The embryos are then permeabilized a second time by placing the embryos at -20°C in 80% acetone/ddH_2_O for 10min, and then washes 2 times for 5 min in PBT. The embryos are then refixed with 4% formaldehyde in PBT for 20min, and washed 5 times for 5min in PBT.

After this, the embryos are washed 2 times with Hb-B (50% formamide, 5x SSC (PH 5.0), 0.1% Tween-20) for 5 min. During the last wash, 200µL per sample of Hb-A (50% formamide, 5x SSC (PH 5.0), 0.1% Tween-20, 100µg/ml denatured Salmon sperm DNA, 50μg/ml Heparin) are placed into separate tubes. The tubes are heated to 80°C for 5min, and then chilled on ice for 5min. The embryos are then divided into the prepared tubes and prehybridized on a rocking thermoblock, at the corresponding hybridization temperature, for 2h.

After 2h, 10-20ng of probe are diluted into 100µL of Hb-A. These diluted probes are then heated to 80°C for 3min and chilled on ice for 5min. The prehybridization solution is then replaced by the new Hb-A with the probe, and the embryos are hybridized on a rocking thermoblock at the corresponding temperature overnight.

The morning after, the embryos are washed twice for 15min and 4 times for 30min in Hb-B at the hybridization temperature. The embryos are then washed 5 times for 5min in PBT. The PBT is replaced with MABT and wash 2 times for 5 min in MABT. DR. JAVIER CASTRO rotates the embryos overnight at 4°C in 200µL MABT (100mM Maleic acid buffer (pH7.5), 150 mM NaCl, 0.1% Tween-20) containing 1:2000 preabsorbed sheep anti-DIG-AP (Roche).

Next morning, the embryos are washed 5 times for 5min and 5 times for 10min in MABT. The last wash is then replaced with DIG-3 and wash twice for 5min.

The embryos are then transferred to glass plates and stained in a DIG-3 (100mM Tris.HCl pH 9.5, 100 mM NaCl, 50mM MgCl_2_) containing 8µL/mL of NBT-BCIP (Sigma-Aldrich).
