# Supplementary material for: Repression of the Hox gene abd-A by ELAV-mediated Transcriptional Interference
Source: PLoS Genet. 2021 Nov 15;17(11):e1009843. doi: 10.1371/journal.pgen.1009843 (PMC8629391; doi:10.1371/journal.pgen.1009843)
Supplement: Attachment — Submitted filename: Castro_et_al._response_letter.docx [file pgen.1009843.s013.docx]

Dear Editors of PLoS Genetics:

Thank you very much for reviewing our manuscript entitled, “Repression of the Hox gene *abd-A* by ELAV-mediated Transcriptional Interference”, by Castro et al, and providing useful comments to improve its quality. We were extremely happy with the positive reviews. Based on the reviewer comments, we have gone through our manuscript and added explanations and clarifications to the text and figures. In one case, one of the reviewers asked if we could see a difference in the level of intergenic transcription between *elav* and *elav, fne* double mutants using our intergenic *in situ* probe. To answer this question, we thought it best to address this question using RT-qPCR (figure 7). Luckily, our collaborator Valerie Hilgers had appropriate resources to quickily perform these experiments. As such, Valerie Hilgers and her student, Judit Carrasco have been added to the list of authors. This data is shown in a new figure (figure 7) and includes a visualization of the transcription occurring between the *iab-8* and *abd-A* genes in *elav*, and *elav, fne* double mutants according to the Hilgers’ lab recently published RNA-seq study (Carrasco et al. 2020) and a quantification the level of this transcription using RT-qPCR at two embryo collection time points. Finally, based on the editor’s suggestion, we changed the wild type image in figure 4 so as not to repeat this image in two figures.

Please find below a copy of the specific points raised by the reviewers and our specific responses to each point, written in italics.

**Response to points of Reviewer 1:**
**…In my opinion two points needs considerations**

1-I feel the de-repression even in the context of elav, fne and mir-iab8 removal (Fig. 1G) does not phenocopy lack of repression induced by cis modifications (Fig. 1F). The authors disregard this difference that yet is apparent. This could be corrected either by considering it, or providing another illustration if this one is not best representing the data obtained. Also, it would be interesting to assess the impact of the elav, fne and mir-iab8 context using the intergenic probe and see if it results in a stronger loss of read through when compared to elav mutation alone (Fig. 6F).

*Indeed, it does seem that there may be a slight difference between the triple mutant and the complete loss of the ncRNA. This is why we state that there is a strong derepression of Abd-A in PS13 rather than a complete derepression. We did not want to judge this too much in the manuscript due to the fact that embryos mutant for elav and elav, fne have abnormal nerve cords. Although the nerve cords look, at a cursory glance, similar to wt nerve cords, we noticed during dissection that they are markedly more fragile. Thus, they might have other differences at the cellular level that we have not characterized. If we examine the main differences between the triple mutant and Fab-8 64, we primarily see that the difference seems to be a few cells located at the posterior end of the parasegment; Cells along the left and right sides seem missing and a few cells, more centrally located, seem less intense. As I said above, the triple mutant embryos have been much harder to dissect. The only CNSs that seem to survive dissection are slightly older, when the posterior CNS starts to contract laterally, we believe this is cause for the lateral differences. Unfortunately, of all of the CNSs that we dissected and survived, this was the youngest embryo we could obtain. It is very similar in age to the other embryos shown, but does have a slight contraction in PS13. On the other hand, the central cells, may be a real difference. We have added a section to the manuscript pointing this out and discussing the possible causes (like the rbp9 gene, a third elav paralogue).*

*We have also added a figure provided by Dr. Valerie Hilgers (now added to the authors) to demonstrate the difference between elav and elav, fne mutants on the read-through transcription of the iab-8 ncRNA. Although, we also performed in situs on elav mutants alone, which shows a lower amount of ectopic abd-A transcript in PS13, we felt that Dr. Hilgers’ RNA-seq experiment would be a better, independent confirmation. Additionally, Dr. Hilgers’ provided an RT-qPCR experiment to verify this change (which was subtle on the RNA seq, due to the fact that the RNA was isolated from whole embryos). These results are shown in Figure 7 and confirm the expected results.*

2- The authors could discuss more explicitly the functional meaning that all except one read through transcript lack the initial AbdA ATG. If this read through transcript allows AbdA expression then how the whole mechanisms of transcriptional interference seems irrelevant to AbdA expression…Is this isoform very weakly expressed ?

*2. This is a very interesting point. As the reviewer mentions, all but one read through transcript would lack the initial ATG. Still, one transcript keeps the ATG. And, although it probably represents a small amount of abd-A transcript relative to the endogenous transcription (see figure 2 in which we see the about of abd-A made from the iab-8 transcript relative the endogenous promoter), one might imagine that its presence would be counter to the function of the transcriptional interference. How does the transcriptional interference work, if the iab-8 transcript might lead to the transcription of the abd-A coding sequence made via another promoter? It turns out that the iab-8 ncRNA exons 1 and 2 contain elements that prevent translation. We first realized this when we placed the coding sequence of mCherry into the endogenous iab-8 ncRNA exon 3. Using these flies, we found no mCherry expression in the CNS, even though the transcript was made, spliced and exported from the nucleus. However, we later learned that iab-8 exons 3-8 are part of second RNA, called MSA that starts from an alternative 1^st^ exon and is expressed in the male accessory gland. When we checked for mCherry expression in the accessory gland, we found robust mCherry fluorescence. This work initial result was published in PLoS Genetics in 2018 (*[*https://doi.org/10.1371/journal.pgen.1007519*](https://doi.org/10.1371/journal.pgen.1007519)*) and continued in an article published in PNAS 2021 (DOI: 10.1073/pnas.2001897118). We have since shown that exons 1 and 2 both contain elements that repress translation on any downstream open reading frame tested. We are in process of writing up these results. For this manuscript, I have discussed the repressive elements in exons 1 and 2 based solely on the published results in order to avoid citing unpublished data.*

**Minor points:**
1- The work strongly relies on previous work of the group to which the authors refers to. However stating what exactly in this previous work support the rational for the experimental strategy used in this MS would facilitate the reading. This is for example striking in the first section of the results part, where the authors indicate in two places that previous work suggested that the repressive element was located towards the 3’ end of the ncRNA (ref14). Summarizing the reason for that for the reader would be helpful.

*We apologize for the confusion. We have gone through the manuscript and added more information to clarify these issues.*

2-Repression seems to occur in the late CNS. Why the RT PCR characterization of the iab-8 ncRNA extension into AbdA was performed starting from 0-24h embryos seems not properly justified. Would the diversity of transcripts characterized be lower if a later and shorter time window was selected?

*In the new figure, the RNA-seq and Q-PCR results were performed on samples taken at different time points and with short collection windows.*

3-In the second section of the result part, the authors indicate that they see a correlation between iab-8 ncRNA extension and abdA repression. It is not clear to me what support that claim ?

*We are sorry if there was a confusion regarding this point. What we mean is that when we reduce/eliminate the iab-8 extension (elav/fne mutants, the inversion and deletions of the promoter) we see increased abd-A expression and when we have mutants that keep the read through transcription (internal deletions), we retain abd-A repression. We have tried to make this clearer in the text.*

4-Also in the second section of the result part, the authors indicate that EGFP reporter construct “almost completely” re-creates the AbdA promoter region…”. The authors could be more specific indicating what is different …

*Again, we are sorry for the confusion. We said “ almost completely” because we were loose with our interpretation of the promoter, as we did not want to imply that our construct is exactly like the wild type abd-A locus. If we consider solely the basal promoter region, we actually perfectly recreate this region. However, our locus differs from a normal abd-A region in the fact that there is a duplication of the promoter (we keep the endogenous abd-A promoter downstream of our reporter). Thus, we thought saying “perfectly recreates” would suggest perfect regulation, something that we have not verified outside of the contexts of this manuscript. Although we believe the regulation is accurate, we must admit that enhancers must now be shared and this might cause a slight problem. We have modified the text to read, “In the reporter line, we fused the EGFP cDNA to the abd-A promoter and the first 140bp of the abd-A 5’UTR. We then integrated this construct (which also contains iab-8 exon 8) into the genomic locus of exon 8 of the iab-8 ncRNA (abdA:GFP:* ***Fig. 4****) to recreate the abd-A promoter region downstream from the endogenous iab-8 ncRNA in the BX-C, but with the EGFP sequence replacing abd-A.” As we speak of intention, there is no need to say “almost completely” as the obvious differences are stated in the text.*

5- Representing in Fig. 1 all the deletions generated and analyzed, and not only the largest one, could be of interest for the community.

*We have added the deletions used in this work and refer to the other deletions tested in previous works (see response to minor point 1) .*

6- The embryo displayed in panel D, Fig.S1 looks much older (all three gut constrictions are formed) than the others (gut not yet compartmentalized). To make sure differences are not due to that, an earlier embryo could be shown.

*Thank you very much for this comment. And while we agree with this sentiment, in principle, we found that the staining on this genotype is very weak but increases with embryonic age. At earlier stages the embryos were unstained and thus we could not be sure the individual embryos were properly permeablized. Since the staining was basically background, we chose an older embryo to image, where some color was visible.*

7- Figure 3: The authors could chose a representation where each transcript is displayed, so to more easily grasp the exact exon content of each of them.

*In fact, we had such a figure in a previous draft of the manuscript and removed it to save space. We have now removed the condensed figure in replace of the expanded one.*

**Reviewer #2:**

**… minor comments**

1. A recent study from the same group (Immarigeon et al., 2021) reported the expression of a secondary cell specific micropeptide from exon-8 of iab-8, with a reproductive function. Given the extension of iab-8 transcript beyond exon-8 in CNS, did authors check for such micropeptide-ORFs in the region of interest in the present study? Can such peptides mediate/participate in the repression and could those be targeted by Elav and/or fne?

*Great question. We have not looked into this, primarily because of a translational repression activity that we discovered in exons 1 and 2 of the iab-8 RNA. This activity is what prevents the MSA micropeptide from being expressed in the CNS (a CDS that is shared between the two transcripts). Further studies have shown that this repressive function represses other reporters placed downstream of them (see response to reviewer 1 point 2). In the case of the transcriptional interference that we see here, it mostly does not come into effect as the starting ATG is removed from most spliced products.*

*Because we have been asked recently, when presenting the translational control work, if this means that the transcriptional interference could all be explained by it being a part of the translation control mechanism, we must say that these two phenomena are separate, as abd-A levels go up when the iab-8 promoter is removed. Thus, transcriptional interference works on transcription from the abd-A promoter and not from transcription coming from the iab-8 promoter.*

2. It would be great if discussion can also dwell upon the functional relevance of such tissue specific repression mechanism in the context of iab-8/abd-A functions reported earlier.

*Thank you for the suggestion. We were not completely sure what you exactly meant by this comment but we have added a couple of sentences about the functional relevance of the repression of abd-A to the introduction. We wanted to interpret the question as asking about the transcriptional interference being used as a mechanism to mediate tissue specific repression, but we were not sure that is what you asked. In any case, we had a very difficult time working in this issue into the discussion because it is largely conjectural on our part. Basically, the point dealt with the fact that factors like fne and rbp9 are not exclusive to the CNS and thus might be expressed in areas of the body where ELAV is not present. As FNE (and maybe RBP9) is able to perform ELAV functions when ELAV is not present (and only when ELAV is not present), we hypothesized that transcriptional elongation and interference could happen in other tissues of the fly. This is something we are looking into at the moment, but do not yet have data to support this.*

3. Certain issues with figures/figure legends need to be fixed: For example, The figure legend for Figure 4 reads as “Each panel shows a Z-projection image made from confocal stacks of the posterior stage 13/14 CNS stained with: anti-ABD-A (in red in A., in grey in B.), anti-GFP (green C.-F.) and EN (blue in A. to show the anterior parasegment boundaries)”. However, Fig. 4A represents schematic while Fig. 4B (red) &C (grey) are the wild type. GFP is seen in Figs. 4D-F. Also EN is seen in Fig.4B but not in 4A. Abd-A-EGFP stained with EN could have been included. Also the relevance of using two wild type figures (red & grey) in this figure is not clear.

*We apologize for the confusing figure legend and thank the reviewer for pointing this out. Indeed, we should have more clearly listed the antibodies in the order they were presented. This has been changed, as has the wt figure, which we changed at the recommendation of the editor (we used a different image that only showns the costaining with EN to mark the parasegment boundaries). We decided to retain the wt image here just for reader convenience, as we, personally, hate to have to flip back and forth among figures to compare staining.*

*Regarding the other points, I am not sure that I understand everything. Perhaps, there was a problem with the version of the figure legend that you received. As the version that we provided to the PLoS Genetics site reads:*

*Figure 4: ABD-A:GFP expression in different genetic contexts.* ***A.*** *A magnification of the abd-A-EGFP reporter construct inserted downstream from exon 8 is displayed in the triangle below the broader genomic context. The resulting construct consists of a duplication of the exon-8-abd-A interval. The EGFP coding sequence starts after 140 bps of the abd-A 5’UTR.* ***B.-F****. Each panel shows a Z-projection image made from confocal stacks of the posterior stage 13/14CNS stained with: anti-ABD-A (in red in A., in grey in B.), anti-GFP (green C.-F.) and EN (blue in A. to show the anterior parasegment boundaries). Genotypes of the embryos are indicated in each panel. Scale bar = 20 μm.*

*In any case, we modified the figure legend to represent the current version. We hope that this is less confusing.*
